# Draft Genome Sequence of Organophosphate-Degrading *Ochrobactrum anthropi* FRAF13

**DOI:** 10.1128/genomeA.00295-16

**Published:** 2016-04-21

**Authors:** Rupa Iyer, Ashish Damania

**Affiliations:** a Center for Life Sciences Technology, College of Technology, University of Houston, Houston, Texas, USA; b Department of Pediatrics, Section of Pediatric Tropical Medicine, Baylor College of Medicine, Houston, Texas, USA

## Abstract

*Ochrobactrum anthropi* FRAF13 was isolated from farmland soil in Jersey Village, Texas. FRAF13 is a bacterial microorganism with broad antibiotic resistance that possesses a number of metal-dependent β-lactam enzymes with secondary phosphotriesterase activity that can initiate the breakdown of organophosphate compounds.

## GENOME ANNOUNCEMENT

*Ochrobactrum anthropi* is a Gram-negative bacterium reported to possess broad-spectrum antibiotic resistance and represents a growing concern in clinical settings as a potential human pathogen in immunocompromised individuals (1). Here, we report a draft genome sequence of a new strain of *O. anthropi*, isolated from farmland soil through an Environmental Sampling Research Module undertaken by University of Houston biotechnology undergraduates (2). Designated *O. anthropi* FRAF13, this microorganism is closely related to *Ochrobactrum anthropi* ATCC 49188 with a similar antibiotic resistance gene complement (3). *O. anthropi* FRAF13 is a particularly efficient organophosphate degrader, characterized for its capacity to degrade the insecticides ethyl paraoxon and methyl parathion to *p*-nitrophenol, possibly through the secondary action of one or more of its many mettalo-β-lactamase (MBL) enzymes. As such, this genome may be particularly useful in elucidating the evolution of phosphotriesterase activity in MBLs. Lastly, FRAF13 has also shown some capacity for breaking down the herbicide glyphosate and may harbor a FAD-dependent oxidoreductase similar to the GOX enzyme found in other *Ochrobactrum* spp. (4). The genome sequencing of FRAF13 was performed through Illumina MiSeq paired-end sequencing with a final sequencing coverage of 144.52×. Sequence reads were checked for quality using FastQC (5) and filtered using BBTools (6). Paired-end reads were then assembled into a total of 17 contigs with the Spades version 3.6.2 program (7). Preliminary reference-based annotation using PATRIC (8) was carried out to identify conserved pathways. Final *de novo* annotation was performed with Prokka (9) and the NCBI Prokaryotic Genome Automatic Annotation Pipeline (http://www.ncbi.nlm.nih.gov/genome/annotation_prok). The metabolic pathways of aromatic and heterocyclic compounds were examined through KEGG databases (10). This draft genome of strain FRAF13 consists of a total of 4,538,068 bp encoding for 4,188 putative coding sequences, of which 4,117 are predicted to form hypothetical or known functional proteins. The genome has a GC content of 55.98 and contains 48 tRNA and 4 ncRNA loci.

### Nucleotide sequence accession numbers.

The *Ochrobactrum anthropi* FRAF13 whole-genome shotgun project has the project accession number LSVB00000000. This version of the project (01) has the accession number LSVB01000000, and consists of sequences LSVB01000001 to LSVB01000017.
